# Full genome sequence analysis of a 1-7-4-like PRRSV strain in Fujian Province, China

**DOI:** 10.7717/peerj.7859

**Published:** 2019-10-16

**Authors:** Jiankui Liu, Chunhua Wei, Zhifeng Lin, Wei Xia, Ying Ma, Ailing Dai, Xiaoyan Yang

**Affiliations:** 1 College of Life Sciences, Longyan University, Longyan, China; 2 Fujian Provincial Key Laboratory for the Prevention and Control of Animal Infectious Diseases and Biotechnology, Longyan University, Longyan, China; 3 College of Animal Science, Fujian Agriculture and Forestry University, Fuzhou, China

**Keywords:** PRRSV, Genome sequence, 1-7-4, Molecular characteristic

## Abstract

PRRS virus (PRRSV) has undergone rapid evolution and resulted in immense economic losses worldwide. In the present study, a PRRSV strain named FJ0908 causing high abortion rate (25%) and mortality (40%) was detected in a swine herd in China. To determine if a new PRRSV genotype had emerged, we characterized the genetic characteristics of FJ0908. Phylogenetic analysis indicated that FJ0908 was related to 1-7-4-like strains circulating in the United States since 2014. Furthermore, the ORF5 sequence restriction fragment length polymorphism (RFLP) pattern of FJ0908 was 1-7-4. Additionally, FJ0908 had a 100 aa deletion (aa329–428) within nsp2, as compared to VR-2332, and the deletion pattern was consistent with most of 1-7-4 PRRSVs. Collectively, the data of this study contribute to the understanding of 1-7-4-like PRRSV molecular epidemiology in China.

## Introduction

Porcine reproductive and respiratory syndrome (PRRS) is a global viral swine disease and causing severe economic loss in the global pig industry (Neumann et al., 2005; Zhou & Yang, 2010; Holtkamp et al., 2013; Gao et al., 2017). PRRS virus (PRRSV), the causative agent of PRRS, is a small enveloped virus with positive-sense single-stranded RNA virus belonging to the Arteriviridae family in the Nidovirales order (Benfield et al., 1992; Meulenberg, 2000).

The PRRSV genome is about 15 kb in length and contains ten open reading frames (ORFs), ORF1a, 1b, 2a, 2b, 3, 4, 5a, 5, 6 and 7. ORF1a and ORF1b encode at least 16 non-structural proteins (nsps) (Nsp1α, Nsp1β, Nsp2-6, Nsp2TF, Nsp2N, Nsp7a, Nsp7b and Nsp8-12), while ORF2-ORF7 encode viral structural proteins: GP2a, E (2b), GP3, GP4, GP5a, GP5, M, and N (Meulenberg, 2000; Wu et al., 2005; Firth et al., 2011; Fang & Snijder, 2010; Fang et al., 2012).

PRRSV is characterized by extensive genetic variation. Based on global PRRSV classification systems, type 2 PRRSV strains in China can be classified into lineage 1 (NADC30-like), lineage 3 (QYYZ-like), lineage 5.1 (VR2332-like), and lineage 8.7 (JXA1-like) (Gao et al., 2017; Liu et al., 2017b; Guo et al., 2018). In Fujian Province, one of the largest livestock trading areas in China, herd movements across provinces and national borders are also common. Hence, multiple PRRSV types co-exist in swine herds. Recently, PRRSV isolates of the ORF5 restriction fragment length polymorphism (RFLP) 1-7-4 viruses emerged in America, causing dramatic abortion “storms” in the sow herd (Alkhamis et al., 2016; Van Geelen et al., 2018). Here, we report a genetic and phylogenetic analysis of PRRSV isolate FJ0908 belong to the ORF5 1-7-4 viruses in Fujian Province, China.

## Materials and Methods

### Clinical case

In September 2018, severe reproductive and respiratory disease was observed in piglets in a farm. The affected pigs had respiratory distress, and high abortion rate (25%) and mortality (40%).

### Strain identification and nucleotide sequencing

PRRSV infection was confirmed by Real-time RT-PCR testing of the serum of affected pigs according to the manufacturer’s instructions. The ORF5 sequence RFLP pattern was inferred according to Wesley et al. The complete genomic sequences of FJ0908 were amplified as described previously (Zhang et al., 2018). The PCR products were purified and cloned into pGEM-T Easy according to the manufacturer’s instructions (Promega, Madison, WI, USA) and three recombinant clones of every fragment were sequenced by Ruibo Life Technologies Corporation (Beijing, China).

### Complete genomic sequence analysis

Fifty-four representative type 2PRRSV sequences in GenBank were utilized in phylogenetic analyses (Table 1). Multiplex sequence alignments were performed using CLUSTAL X (version 1.83) and the phylogenetic relationships were assessed by MEGA 6.0 as described (Liu et al., 2017b). The ORF5 sequences were classified according to the global PRRSV classification systems (Shi et al., 2010).

**Table 1:** PRRSV strains used in this study.

No.	Name	GenBank accession no.	Origin	No.	Name	GenBank accession no.	Origin
1	FJ0908	MK202794	China	29	SDSU73	JN654458	USA
2	FJZ03	KP860909	China	30	NADC31	JN660150	USA
3	FJY04	KP860910	China	31	NB/04	FJ536165	China
4	CH-1a	AY032626	China	32	BJ-4	AF331831	China
5	JA142	AY424271	USA	33	MN184A	DQ176019	USA
6	VR-2332	U87392	USA	34	MN184B	DQ176020	USA
7	JXA1-R	R FJ548855	China	35	HENNAN-XINX	KF611905	China
8	JXA1	EF112445	China	36	NADC30	JN654459	USA
9	FJFS	KP998476	China	37	JL580	KR706343	China
10	PA8	AF176348	Canadia	38	CHsx1401	KP861625	China
11	Em2007	EU262603	China	39	ISU30	KT257977	USA
12	GM2	JN662424	China	40	MN184C	EF488739	USA
13	QYYZ	JQ308798	China	41	NC/2014/ISU-3	MF326990	USA
14	HB-1(sh)/2002	AY150312	China	42	LV	M96262	Netherlands
15	HB-2(sh)/2002	AY262352	China	43	NC/2014/ISU-4	MF326991	USA
16	HUN4	EF635006	China	44	IN/2014/ISU-5	MF326992	USA
17	OH/2014/ISU-6	MF326993	USA	45	Ingelvac ATP	DQ988080	USA
18	NC/2015/ISU-11	MF326998	USA	46	RespPRRS MLV	AF066183	USA
19.	IA/2015/ISU-13	MF327000	USA	47	NC/2015/ISU-12	MF326999	USA
20	LNWK130	MG913987	China	48	IA/2015/NADC35	MF326986	USA
21	NCV-13	KX192112	USA	49	IA/2015/NADC36	MF326987	USA
22	NCV-23	KX192116	USA	50	IA/2015/ISU-10	MF326997	USA
23	NCV-25	KX192118	USA	51	IA/2014/NADC34	MF326985	USA
24	OH/2014/ISU-7	MF326994	USA	52	LNWK96-CN	MG860516	China
25	IA/2014/ISU-8	MF326995	USA	53	NCV-21	KX192115	USA
26	IA/2015/NADC36	MF326987	USA	54	IA/2015/ISU-9	MF326996	USA
27	FJSD	KP998474	China	55	HUB1	EF075945	China
28	IA/2014/ISU-2	MF326989	USA	56	IA/2015/ISU-14	MF327001	USA

Recombination events were detected using Simplot v 3.5.1 and the boot scanning analysis was performed with a 200-bp window, sliding along the genome alignments with a step size of 20 bp.

## Results

### Complete genomic sequence analysis

The genomes of FJ0908 (GenBank accession no. MK202794) was 15,112 nt in length, excluding the poly (A) tail at the 3′ end, 300 nt shorter than the genome of the prototypic VR2332. Genome alignments revealed that FJ0908 shared 83.6% identity with JXA1, 84.7% with VR2332, 82.2% with QYYZ, 86.3% with NADC30, 97.3–97.6% with 1-7-4 PRRSV family (IA/2014/NADC34, IA/2015/NADC35) and 98.7% with LNWK130 (Table 2).

**Table 2:** Detailed comparison of the full-length genomes of FJ0908 to other PRRSV reference strains.

	VR2332	BJ-4	JXA1	HuN4	FJFS	QYYZ	NADC30	ISU30	LNWK96	IA/2014/NADC34	IA/2015/NADC35	LNWK130
	Sublineage 5.1		Sublineage 8.7		Lineage 3		Sublineage 1.8			Sublineage 1.5		
	Pairwise % Identity to FJ0908 (nt/aa)											
Nucleotides												
Complete genome	84.7	84.6	83.6	83.6	82.2	82.2	86.3	89.4	96.2	97.6	97.5	98.7
5′UTR	93.6	93.6	92.5	92.5	91.0	91.5	95.7	92.9	96.3	97.9	97.3	97.9
ORF1a	82.0	81.9	80.6	80.7	78.6	78.6	82.8	87.8	96.3	97.2	97.2	98.0
ORF1b	86.6	86.6	86.2	86.2	85.8	85.6	88.8	86.2	98.2	98.2	98.1	99.2
ORF2-7	87.2	87.1	85.9	86.0	84.9	84.9	89.4	88.3	93.1	97.6	97.2	99.6
3′UTR	92.7	92.7	90.0	90.1	86.1	88.7	95.4	91.5	95.9	98.0	98.0	98.0
nt 1–760	90.0	89.9	88.1	88.4	86.7	89.1	92.6	94.8	95.4	98.8	98.6	97.1
nt 760–1,300	80.4	80.4	80.6	80.6	78.3	77.2	82.0	94.3	93.9	92.8	9.26	94.4
Amino acids												
NSP1α	94.6	95.2	97.0	97.0	93.4	96.4	95.8	96.4	97.0	98.8	98.8	98.2
NSP1β	78.8	78.8	77.4	77.9	78.3	77.0	79.7	91.7	94.0	91.7	91.7	94.5
NSP2	67.0	67.0	66.5	66.7	63.3	64.6	71.4	72.1	92.7	94.5	94.7	96.4
NSP3	91.3	91.3	89.9	90.1	87.7	87.7	91.7	91.9	97.5	97.8	97.5	98.7
NSP4	93.1	93.1	95.1	95.1	92.6	93.1	92.6	96.6	98.5	99.5	99.5	99.0
NSP5	88.8	88.8	89.4	89.4	90.0	89.4	87.6	94.7	97.6	98.8	98.8	98.8
NSP6	87.5	87.5	93.8	93.8	93.8	93.8	87.5	87.5	93.8	93.8	93.8	93.8
NSP7	90.3	89.2	87.3	87.3	86.5	84.6	89.2	95.4	98.8	98.8	98.8	99.2
NSP8	93.3	93.3	93.3	93.3	88.9	93.3	95.6	100	100	100	100	97.8
NSP9	95.8	95.5	96.1	95.8	93.9	94.8	95.8	97.2	98.3	98.9	98.9	99.1
NSP10	95.0	95.0	94.6	95.2	92.3	94.1	98.4	98.2	99.8	99.8	99.5	99.5
NSP11	95.1	95.5	96.0	96.0	94.6	94.6	95.1	94.6	100	99.6	99.6	100
NSP12	90.3	90.2	90.9	90.9	92.2	91.5	89.0	98.0	99.3	98.7	98.7	100
ORF2a/GP2	85.5	86.3	85.9	85.5	80.1	82.8	83.6	84.0	97.7	98.4	96.9	99.6
ORF2b/E	87.8	89.0	86.3	86.3	80.8	91.8	90.4	90.4	97.3	100	98.6	98.6
ORF3/GP3	80.7	80.7	79.9	79.5	80.7	79.9	81.5	81.9	89.4	94.5	93.3	99.2
ORF4/GP4	86.0	84.8	86.5	88.8	88.8	86.5	94.4	92.1	91.6	94.4	94.4	99.4
ORF5/GP5	87.0	87.0	86.0	86.5	85.5	85.5	90.5	88.0	90.0	97.5	97.5	98.5
ORF5a	91.3	91.3	84.8	84.8	84.8	87.0	95.7	93.5	95.7	100	100	97.8
ORF6/M	95.4	94.8	93.7	93.7	95.4	94.8	94.8	93.7	95.4	97.7	97.7	100
ORF7/N	91.1	90.2	90.2	90.2	87.8	87.8	95.9	93.5	9.19	96.7	95.1	99.2

The results also showed that 5′-UTR, ORF1a, ORF1b, ORFs 2-7 and the 3′-UTR of FJ0908 shared 97.2–98.2% nucleotide homology with 1-7-4 PRRSV family (IA/2014/NADC34, IA/2015/NADC35), which was higher than the homology shared with other representative strains, indicating that FJ0908 strain belonged to 1-7-4 PRRSV. ORF1a and ORF1b encode 16 nsps of PRRSV, Nsp1β and Nsp2 are the most variable protein products among these nsps (Table 2). ORFs 2 to 7 encode the PRRSV structural proteins, among these structural proteins, GP2, GP3, GP4, GP5a and GP5 exhibited the most variance (Table 2).

### Amino acid analysis of Nsp2

Nsp2 contains different deletions and insertions, as compared to VR2332 and is the most variable protein in PRRSV genome Liu et al., 2017b; Li et al., 2011). Strikingly, the nsp2 gene of the FJ0908 was 2,640 nt in length and encoded 880 aa, with a 100 aa deletion (aa329–428) within nsp2, as compared to VR2332, and the deletion pattern was consistent with most of 1-7-4 PRRSVs.

### Antigenic analysis of GP2-GP5

The antigenic regions (ARs) and glycosylation sites within the GP2, GP3, GP4, and GP5 proteins of FJ0908 were predicted and compared to OH/2014/ISU-7 IA/2014/ISU-8, IA/2014/NADC34, IA/2015/NADC35, IA/2015/NADC36, LNWK96, LNWK130, NV-21, NV-25, VR2332, CH-1a, JXA1 and NADC30.

In GP2, two antigenic regions (AR 41–55 and AR123–135) were confirmed in type 2 PRRSV (De Lima et al., 2006). The predicted AR at aa 41–55 were highly conserved and no aa substitution was detected in AR123–135. In GP3, four predicted antigenic regions (AR32–46, AR 51–105, AR 111–125, and AR 137–159) were proven (De Lima et al., 2006; Zhou et al., 2006; Wang et al., 2014). The AR comprising aa32–46, aa51–105, aa111–125, and aa137–159 of FJ0908 was most similar to 1-7-4 representative PRRSVs including LNWK130, but differed from VR2332, CH-1a, JXA1 and NADC30. GP4 has one predicted AR at aa51–65 (De Lima et al., 2006). FJ0908 had 1-2 aa substitutions as compared to the 1-7-4 representative PRRSVs, but had 2-6 aa substitutions as compared to VR2332, CH-1a, JXA1 and NADC30 and LNWK96. Additionally, no aa substitution was detected in AR51–65 between FJ0908 and LNWK130. The putative glycosylation sites in GP2-4 were completely conserved among the investigated strains, except for GP2 of isolate IA/2015/NADC36 that had one substitution N^184^D.

GP5 is the major envelope protein encoded by ORF5 and is the most variable PRRSV protein. Sequences alignments of GP5 revealed that FJ0908 shares 87.0%, 86.0–86.5%, 85.5%, 88.0–90.5% and 97.5% amino acid identity with VR-2332-like (VR2332 and BJ-4), JXA1-like (JXA1 and HuN4), QYYZ-like (FJFS and QYYZ), NADC30-like strains (NADC30 and ISU30), and 1-7-4-like (IA/2014/NADC34, IA/2015/NADC35), respectively (Table 2). Furthermore, the restriction sites analysis showed that the ORF5 RFLP of FJ0908 has the same 1-7-4 pattern [1 (MluI = 0 sites), 7 (HindII = nt 88, 219, 360), 4 (SacII = nt 24, 555)] (Fig. S1).

GP5 has six ARs (AR1–15, AR27–35, AR37–51, AR149–156, AR166–181, and AR192–200) (De Lima et al., 2006; Zhou et al., 2009). In GP5, the N-terminus ARs (AR1-15 and AR27-35) were very variable among all strains and only three aa substitutions (K^4^N, Q^13^R and L^15^P) were found in the two antigenic regions between FJ0908 and LNWK130, whereas the other four ARs (AR 37–51, AR 149–156 , AR 166–181, and AR 192–200) were conserved. GP5 had differential predicted N-glycosylation among PRRSV strains. FJ0908 possessed five predicted sites (N32, N44, N51, N57 and N59). Although N-glycosylation at N57 was not a novel finding, N57 was detected in most of the 1-7-4 sequences. The results also revealed that FJ0908 had the same N-glycosylation pattern as LNWK96 and LNWK130.

### Phylogenetic analysis

**Figure 1:**
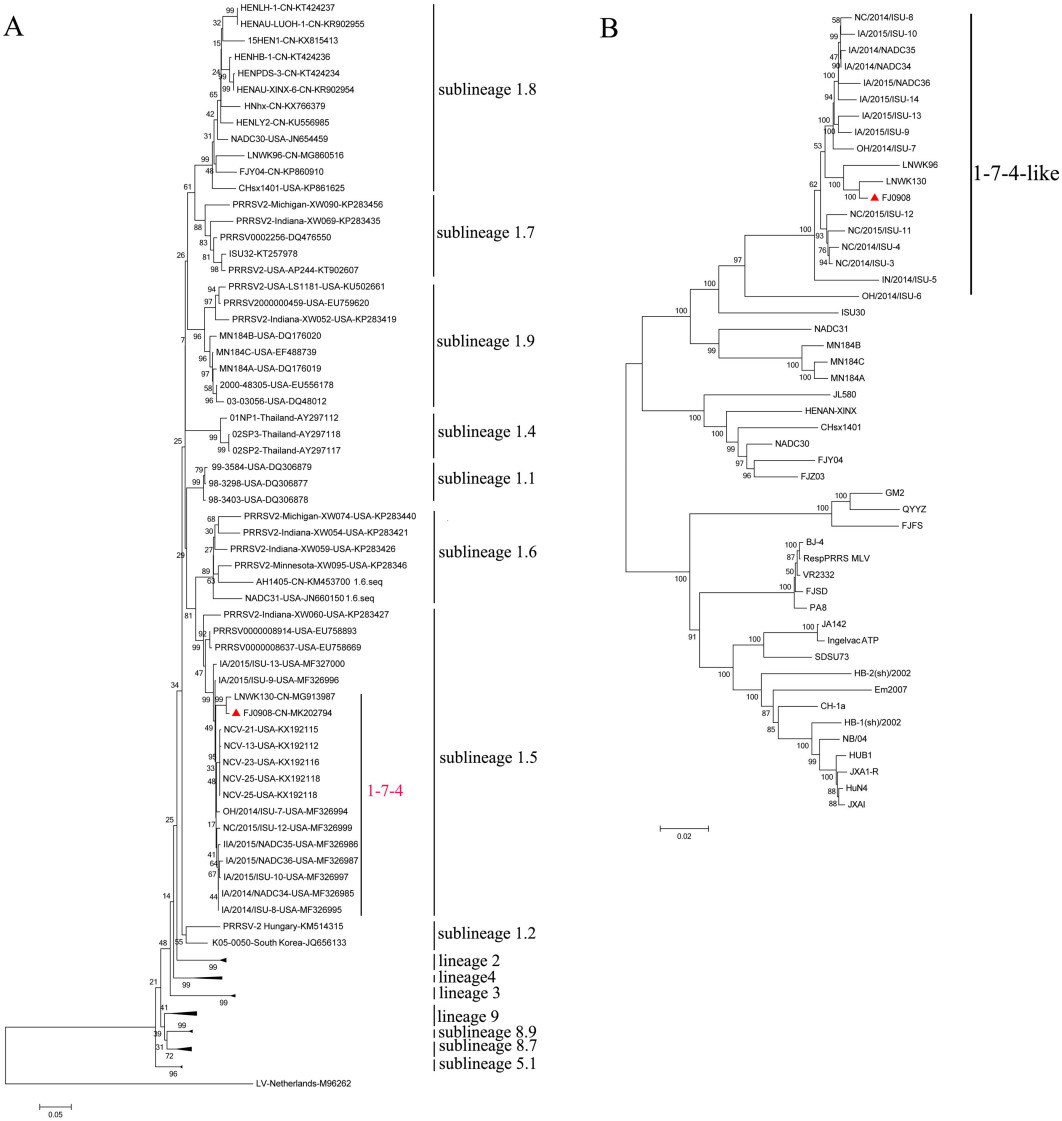
Phylogenetic tree based on the ORF5 genes (A) and full length (B) of the FJ0908 and reference viruses. Reliability of the tree was assessed by bootstrap analysis of 1,000 replications. Our representative isolate FJ0908 were marked with the red triangle (▴). Lineage 1 PRRSVs are divided into nine sublineages.

PRRSV ORF5 is the most variable and has been used as a marker of PRRSV genetic variability. Based on global PRRSV classification systems, type 2 PRRSV was divided into nine monophyletic lineages (1-9) and lineage1 was further classified into nine sublineages (1.1–1.9) (Shi et al., 2010; Guo et al., 2018). The ORF5-based phylogenetic tree showed that FJ0908, as well as 1-7-4 isolates including LNWK130, were clustered in sublineage 1.5 (Fig. 1A). Whole genome phylogenetic analysis also indicated that FJ0908 was most closely related to a genetic cluster in 1-7-4-like lineage 1 (Fig. 1B).

**Figure 2:**
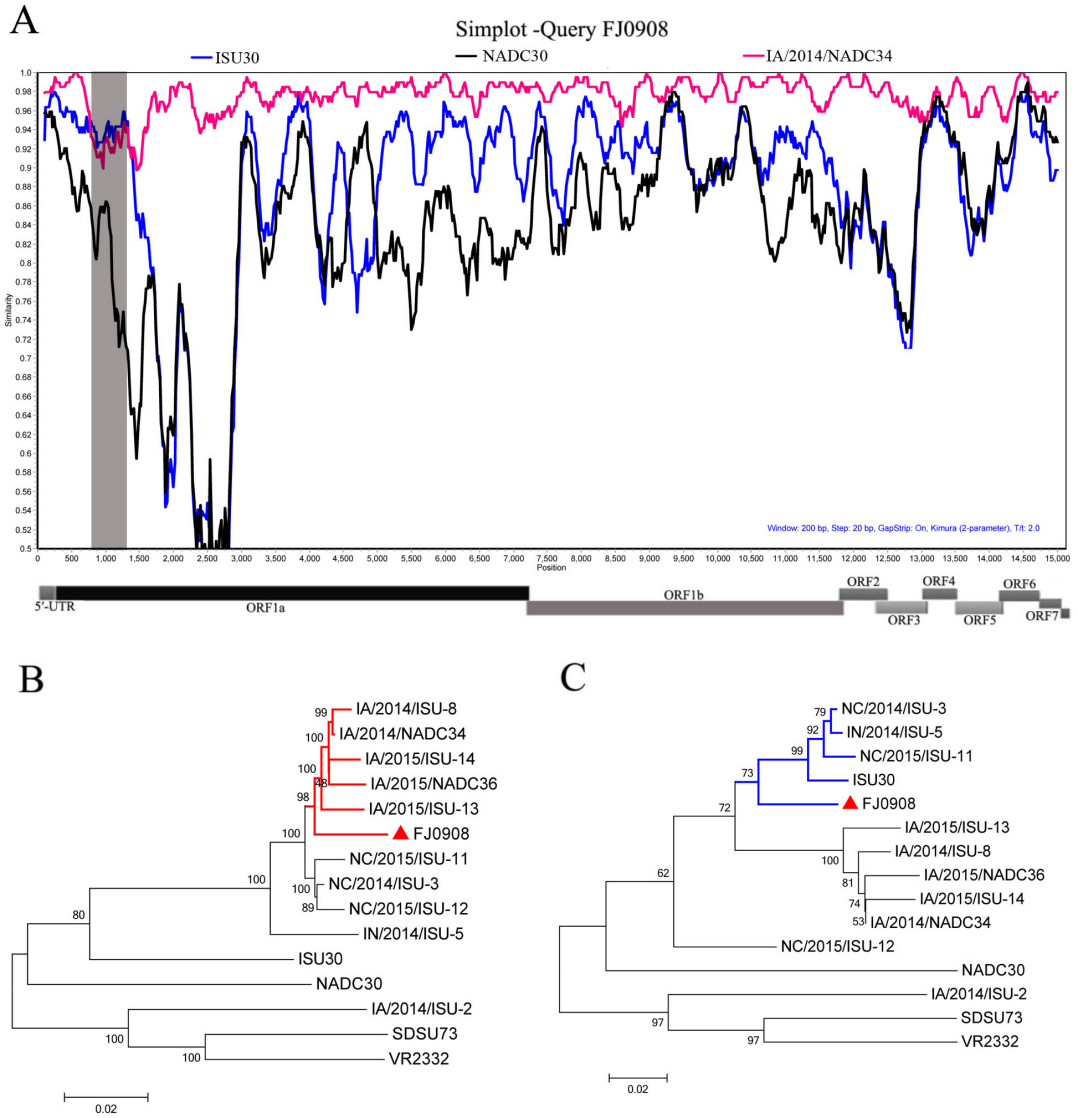
Characterization of the supported recombinant events between FJL0908 and representative PRRSV lineages. (A) Similarity plot and bootscan analyses of FJ0908 by SimPlot. The *y*-axis indicates the percentage similarity between the parental sequences and the query sequence. Phylogenetic trees based on major parental regions (nt 1-760 and nt 1301-15534) (B) and the minor parental region (nt 760–1300) (C). The major parental group (1-7-4 viruses, reference strain IA/2014/NADC34) is shown in red, while the minor parental groups (reference strain ISU30) are shown in blue, respectively.

### Recombination analysis

To test for possible recombinant events within FJ0908 strain, we performed recombinant detection using SimPlot v3.5.1 software. From the similarity plot, two recombination breakpoints within the FJ0908 genome were identified, which were located in Nsp1 (nt 760 and nt 1,300) (Fig. 2A). To further confirm the putative recombination events, phylogenetic trees for each of the sequence regions identified during the analysis were generated, we identified two recombination breakpoints located in nsp1 (nt 760 and nt 1,300) (Figs. 2B and 2C). The two breakpoints separated the genome of FJ0908 into 3 regions. For FJ0908, the region between the breakpoints (nt 760–1,300) is closely related to ISU30 strain, the two regions between the breakpoints (nt 1–759 and nt 1,301–15,534) are closely related to IA/2014/NADC34. Collectively, the above results suggested that FJ0908 derived from recombination between IA/2014/NADC34 and ISU30 (Fig. 2). Moreover, LNWK130 strain (1-7-4-like PRRSV) firstly identified in Liaoning Province, China was also deriving from the recombination of 1-7-4 isolates and ISU30 (Zhang et al., 2018). However, the recombination pattern was different between LNWK130 and FJ0908. For LNWK130, the two breakpoints (nt 760–1,300 and nt 1–759) are closely related to ISU30 strain, one breakpoints (nt 1301–15534) is closely related to IA/2014/NADC34. Additionally, sequences alignments of the recombination region (nt 1-759) revealed that FJ0908 shares 98.8%, 94.8% and 92.6% nucleotide identity with IA/2014/NADC34, ISU30 and NADC30, respectively, in contrast, LNWK130 shares 95.8%, 95.9% and 93.1% nucleotide identity with IA/2014/NADC34, ISU30, and NADC30, respectively. Sequences alignments of the recombination region (nt 760-1,300) revealed that FJ0908 shares 92.8%, 94.3% and 82.0% nucleotide identity with IA/2014/NADC34, ISU30 and NADC30, respectively, similarly, LNWK130 shares 89.4%, 93.0% and 80.2% nucleotide identity with IA/2014/NADC34, ISU30, and NADC30, respectively. For all of the nsp sequences and structural proteins, the most variable regions were found in nsp1β, nsp3, nsp5, nsp6, nsp8, GP3 and GP5 between FJ0908 and LNWK130.

## Discussion

PRRSV causes major economic losses in swine industry since 1990s. Notably, PRRSV continues to expand its genetic diversity. According to Shi et al. (2010), type 2 PRRSV was classified into nine monophyletic lineages based on ORF5 and extensive genetic variation exists among strains within each lineage. It is hard to define the PRRSV homologous, heterologous virus and pathogenic biotype only focused on single gene analysis. To classify and infer the likely pathogenic biotype, RFLP patterns of ORF5 for type 2 strains is standard approach for veterinarians (Wesley et al., 1998). The RFLP pattern 1-7-4 emerged in the US and has become prevalent since 2014, this nomenclature has been associated with severe disease in herds leading to significant economic losses (Van Geelen et al., 2018). In the present study, FJ0908 was isolated in a farm with high abortion rate and mortality in sows, the restriction sites analysis revealed that the ORF5 RFLP of FJ0908 has the 1-7-4 pattern.

Comparison to PRRS sequences in GenBank indicated FJ0908 belonged to 1-7-4-like PRRSV. The genomic regions with the highest variation were found in Nsp1β, Nsp2, ORF2, ORF3, ORF4, ORF5a and ORF5, the lowest variation were found in Nsp1α, Nsp8–12, and ORF6 (Table 2). FJ0908 had 100 aa deletions within Nsp2 (corresponding to position 328–427 of VR2332 nsp2), as compared to the reference strain VR2332, and the deletion pattern was consistent with 1-7-4 viruses.

The pathogenesis of PRRSV has been linked to the N-glycosylation motifs at certain sites of GP2-GP5 by acting as a glycan shield against minimizing the viral neutralizing antibody response (Wissink et al., 2004; Ansari et al., 2006; Faaberg et al., 2006; Das et al., 2011; Delisle et al., 2012; Wei et al., 2012). Many reports have also suggested that N-glycosylation motifs in GP5 of PRRSV is important for viral infectivity and viral immune evasion (Wissink et al., 2004; Ansari et al., 2006; Jiang et al., 2007; Delisle et al., 2012; Wei et al., 2012). In the present study, GP5 contained five predicted N-glycan motifs in FJ0908: N32, N44, N51, N57 and N59. More interestingly, the Chinese strains LNWK96, LNWK130 and FJ0908 have an additional N-glycan at 59 compared to 1-7-4 isolates in the United States.

Recombination may play an important mechanism in generating genetic diversity in PRRSV (Liu et al., 2011; Murtaugh et al., 2010). Most of the 1-7-4 PRRSV isolates may be most potentially derived from different recombination patterns occurring among the local strains in the United States (Van Geelen et al., 2018). Recently, 1-7-4-like PRRSV strains, LNWK130 isolated in Liaoning Province, China was reported to originate from recombination events between IA/2014/NADC34 and ISU30. Currently, recombination events involving NADC30-like PRRSV strains and other PRRSV strains frequently occurred in China (Zhao et al., 2015; Zhang et al., 2016; Bian et al., 2017; Liu et al., 2017a; Liu et al., 2017c; Zhao et al., 2017; Wang et al., 2018; Zhou et al., 2018; Liu et al., 2019). To test for possible recombinant events within FJ0908 strain, we performed recombinant detection using SimPlot v3.5.1 software. Recombination analysis performed with the available full-length genome sequences revealed FJ0908 maybe originate from recombination events between IA/2014/NADC34 and ISU30. Although FJ0908 and LNWK130 maybe drive from recombination events between IA/2014/NADC34 and ISU30, the recombination pattern of two strains were different. Two recombination breakpoints were identified in nsp1 (nt 760 and nt 1,300) in FJ0908 strain, whereas one recombination breakpoint in nsp2 (nt 1480) in LNWK130, suggesting the ancestor of FJ0908 and LNWK130 were most probably transported from different region of United States.

In conclusion, 1-7-4-like PRRSV was also detected in Fujian Province of China besides Liaoning Province. Therefore, effective strategy should be taken to control 1-7-4-like PRRSV and to monitor herd movements.

## Conclusion

In summary, we thoroughly analyzed a new sublineage of PRRSV strain FJ0908 isolated from Fujian Province, China on the basis of a comprehensive study with the full-length genome. These novel sublineage 1.5 virus is closely related to the ORF5 RFLP 1-7-4 strains. Phylogenetic and molecular evolutionary analyses indicated that FJ0908 originated from a natural recombination event between IA/2014/NADC34 and ISU30. Our data enhance our understanding of the PRRSV evolution in China.

##  Supplemental Information

10.7717/peerj.7859/supp-1 Figure S1 RFLP pattern of digestion by MluI, HindII and SacII

10.7717/peerj.7859/supp-2 Supplemental Information 2 Porcine reproductive and respiratory syndrome virus strain FJ0908, complete genome

## Additional Information and Declarations

### DNA Deposition

The following information was supplied regarding the deposition of DNA sequences: The sequence is available at GenBank: MK202794.

### Data Availability

The following information was supplied regarding data availability: The sequence is available as a Supplemental File and at GenBank.

## References

[ref-1] Alkhamis MA, Perez AM, Murtaugh MP, Wang X, Morrison RB (2016). Applications of Bayesian phylodynamic methods in a recent U.S. Porcine Reproductive and Respiratory Syndrome Virus outbreak. Frontiers in Microbiology.

[ref-2] Ansari IH, Kwon B, Osorio FA, Pattnaik AK (2006). Influence of N-linked glycosylation of porcine reproductive and respiratory syndrome virus GP5 on virus infectivity, antigenicity, and ability to induce neutralizing antibodies. Journal of Virology.

[ref-3] Benfield DA, Nelson E, Collins JE, Harris L, Goyal SM, Robison D, Christianson WT, Morrison RB, Gorcyca D, Chladek D (1992). Characterization of swine infertility and respiratory syndrome (SIRS) virus (isolate ATCC VR- 2332). Journal of Veterinary Diagnostic Investigation.

[ref-4] Bian T, Sun Y, Hao M, Zhou L, Ge X, Guo X, Han J, Yang H (2017). A recombinant type 2 porcine reproductive and respiratory syndrome virus between NADC30-like and a MLV-like: genetic characterization and pathogenicity for piglets. Infection Genetics and Evolution.

[ref-5] Das PB, Vu HL, Dinh PX, Cooney JL, Kwon B, Osorio FA, Pattnaik AK (2011). Glycosylation of minor envelope glycoproteins of porcine reproductive and respiratory syndrome virus in infectious virus recovery, receptor interaction, and immune response. Virology.

[ref-6] De Lima M, Pattnaik AK, Flores EF, Osorio FA (2006). Serologic marker candidates identified among B-cell linear epitopes of nsp2 and structural proteins of a North American strain of porcine reproductive and respiratory syndrome virus. Virology.

[ref-7] Delisle B, Gagnon CA, Lambert ME, D’Allaire S (2012). Porcine reproductive and respiratory syndrome virus diversity of Eastern Canada swine herds in a large sequence dataset reveals two hypervariable regions under positive selection. Infection Genetics and Evolution.

[ref-8] Faaberg KS, Hocker JD, Erdman MM, Harris DL, Nelson EA, Torremorell M, Plagemann PG (2006). Neutralizing antibody responses of pigs infected with natural GP5 N-glycan mutants of porcine reproductive and respiratory syndrome virus. Viral Immunology.

[ref-9] Fang Y, Snijder EJ (2010). The PRRSV replicase: exploring the multifunctionality of an intriguing set of nonstructural proteins. Virus Resarch.

[ref-10] Fang Y, Treffers EE, Li Y, Tas A, Sun Z, Vander Meer Y, De Ru AH, Van Veelen PA, Atkins JF, Snijder EJ, Firth AE (2012). Efficient-2 frameshifting by mammalian ribosomes to synthesize an additional arterivirus protein. Proceedings of the National Academy of Sciences of the United States of America.

[ref-11] Firth AE, Zevenhoven-Dobbe JC, Wills NM, Go YY, Balasuriya UB, Atkins JF, Snijder EJ, Posthuma CC (2011). Discovery of a small arterivirus gene that overlaps the GP5 coding sequence and is important for virus production. Journal of General Virology.

[ref-12] Gao JC, Xiong JY, Ye C, Chang XB, Guo JC, Jiang CG, Zhang GH, Tian ZJ, Cai XH, Tong GZ, An TQ (2017). Genotypic and geographical distribution of porcine reproductive and respiratory syndrome viruses in mainland China in 1996-2016. Veterinary Microbiology.

[ref-13] Guo Z, Chen XX, Li R, Qiao S, Zhang G (2018). The prevalent status and genetic diversity of porcine reproductive and respiratory syndrome virus in China: a molecular epidemiological perspective. Virology Journal.

[ref-14] Holtkamp DJ, Kliebenstein JB, Neumann EJ, Zimmerman JJ, Rotto HF, Yoder TK, Wang C, Yeske PE, Mowrer CL, Haley CA (2013). Assessment of the economic impact of porcine reproductive and respiratory syndrome virus on United States pork producers. Journal of Swine Health and Production.

[ref-15] Jiang WM, Jiang P, Wang XL, Li YF, Wang XW, Du Y (2007). Influence of porcine reproductive and respiratory syndrome virus GP5 glycoprotein N linked glycans on immune responses in mice. Virus Genes.

[ref-16] Li B, Fang L, Guo X, Gao J, Song T, Bi J, He K, Chen H, Xiao S (2011). Epidemiology and evolutionary characteristics of the porcine reproductive and respiratory syndrome virus in China between 2006 and 2010. Journal of Clinical Microbiology.

[ref-17] Liu D, Zhou R, Zhang J, Zhou L, Jiang Q, Guo X, Ge X, Yang MH (2011). Recombination analyses between two strains of porcine reproductive and respiratory syndrome virus *in vivo*. Virus Research.

[ref-18] Liu J, Wei C, Lin Z, Fan J, Xia W, Dai A, Yang X (2019). Recombination in lineage 1, 3, 5 and 8 of porcine reproductive and respiratory syndrome viruses in China. Infection Genetics and Evolution.

[ref-19] Liu J, Zhou X, Zhai J, Li B, Wei C, Dai A, Yang X, Luo M (2017a). Emergence of a novel highly pathogenic porcine reproductive and respiratory syndrome virus in China. Transboundary and Emerging Diseases.

[ref-20] Liu J, Zhou X, Zhai J, Li B, Wei C, Dai A, Yang X, Luo M (2017b). Genetic diversity and evolutionary characteristics of type 2 porcine reproductive and respiratory syndrome virus in southeastern China from 2009 to 2014. Archives of Virology.

[ref-21] Liu J, Zhou X, Zhai J, Wei C, Dai A, Yang X, Luo M (2017c). Recombination in JXA1-R vaccine and NADC30-like strain of porcine reproductive and respiratory syndrome viruses. Veterinary Microbiology.

[ref-22] Meulenberg JJ (2000). PRRSV, the virus. Veterinary Research.

[ref-23] Murtaugh MP, Stadejek T, Abrahante JE, Lam TT, Leung FC (2010). The everexpanding diversity of porcine reproductive and respiratory syndrome virus. Virus Research.

[ref-24] Neumann EJ, Kliebenstein JB, Johnson CD, Mabry JW, Bush EJ, Seitzinger AH, Green AL, Zimmerman JJ (2005). Assessment of the economic impact of porcine reproductive and respiratory syndrome on swine production in the United States. Journal of the American Veterinary Medical Association.

[ref-25] Shi M, Lam TT, Hon CC, Murtaugh MP, Davies PR, Hui RK, Li J, Wong LT, YipC W, Jiang JW, Leung FC (2010). Phylogeny-based evolutionary, demographical, and geographical dissection of North American type 2 porcine reproductive and respiratory syndrome viruses. Journal of Virology.

[ref-26] Van Geelen AG, Anderson TK, Lager KM, Das PB, Otis NJ, Montiel NA, Miller LC, Kulshreshtha V, Buckley AC, Brockmeier SL, Zhang J, Gauger PC, Harmon KM, Faaberg KS (2018). Porcine reproductive and respiratory disease virus: Evolution and recombination yields distinct ORF5 RFLP 1-7-4 viruses with individual pathogenicity. Virology.

[ref-27] Wang L, Wan B, Guo Z, Qiao S, Li R, Xie S, Chen XX, Zhang G (2018). Genomic analysis of a recombinant NADC30-like porcine reproductive and respiratory syndrome virus in China. Virus Genes.

[ref-28] Wang X, Dang R, Liu W, Yang Z, Du E, Zhang S (2014). Antigenic characteristics of glycosylated protein 3 of highly pathogenic porcine reproductive and respiratory syndrome virus. Virus Research.

[ref-29] Wei Z, Lin T, Sun L, Li Y, Wang X, Gao F, Liu R, Chen C, Tong G, Yuan S (2012). N-linked glycosylation of GP5 of porcine reproductive and respiratory syndrome virus is critically important for virus replication *in vivo*. Journal of Virology.

[ref-30] Wesley RD, Mengeling WL, Lager KM, Clouser DF, Landgraf JG, Frey ML (1998). Differentiation of a porcine reproductive and respiratory syndrome virus vaccine strain from North American field strains by restriction fragment length polymorphism analysis of ORF5. Journal of Veterinary Diagnostic Investigation.

[ref-31] Wissink EH, Kroese MV, Maneschijn-Bonsing JG, Meulenberg JJ, Van Rijn PA, Rijsewijk FA, Rottier PJ (2004). Significance of the oligosaccharides of the porcine reproductive and respiratory syndrome virus glycoproteins GP2a and GP5 for infectious virus production. Journal of General Virology.

[ref-32] Wu WH, Fang Y, Rowland RR, Lawson SR, Christopher-Hennings J, Yoon KJ, Nelson EA (2005). The 2b protein as a minor structural component of PRRSV. Virus Research.

[ref-33] Zhang HL, Zhang WL, Xiang LR, Leng CL, Tian ZJ, Tang YD, Cai XH (2018). Emergence of novel porcine reproductive and respiratory syndrome viruses (ORF5 RFLP 1-7-4 viruses) in China. Veterinary Microbiology.

[ref-34] Zhang Q, Jiang P, Song Z, Lv L, Li L, Bai J (2016). Pathogenicity and antigenicity of a novel NADC30-like strain of porcine reproductive and respiratory syndrome virus emerged in China. Veterinary Microbiology.

[ref-35] Zhao H, Han Q, Zhang L, Zhang Z, Wu Y, Shen H, Jiang P (2017). Emergence of mosaic recombinant strains potentially associated with vaccine JXA1-R and predominant circulating strains of porcine reproductive and respiratory syndrome virus in different provinces of China. Virology Journal.

[ref-36] Zhao K, Ye C, Chang XB, Jiang CG, Wang SJ, Cai XH, Tong GZ, Tian ZJ, Shi M, An TQ (2015). Importation and recombination are responsible for the latest emergence of highly pathogenic porcine reproductive and respiratory syndrome virus in China. Journal of Virology.

[ref-37] Zhou L, Kang R, Zhang Y, Ding M, Xie B, Tian Y, Wu X, Zuo L, Yang X, Wang H (2018). Whole genome analysis of two novel type 2 porcine reproductive and respiratory syndrome viruses with complex genome recombination between lineage 8, 3, and 1 strains identified in Southwestern China. Viruses.

[ref-38] Zhou L, Yang H (2010). Porcine reproductive and respiratory syndrome in China. Virus Research.

[ref-39] Zhou YJ, An TQ, He YX, Liu JX, Qiu HJ, Wang YF, Tong GZ (2006). Antigenic structure analysis of glycosylated protein 3 of porcine reproductive and respiratory syndrome virus. Virus Research.

[ref-40] Zhou YJ, Yua H, Tian ZJ, Liu JX, An TQ, Peng JM, Li GX, Jiang YF, Cai XH, Xue Q, Wang M, Wang YF, Tong GZ (2009). Monoclonal antibodies and conserved antigenic epitopes in the C terminus of GP5 protein of the North American type porcine reproductive and respiratory syndrome virus. Veterinary Microbiology.

