# Supplementary figures and images for: Full genome sequence analysis of a 1-7-4-like PRRSV strain in Fujian Province, China
Source: PeerJ. 2019 Oct 16;7:e7859. doi: 10.7717/peerj.7859 (PMC6800524; doi:10.7717/peerj.7859)

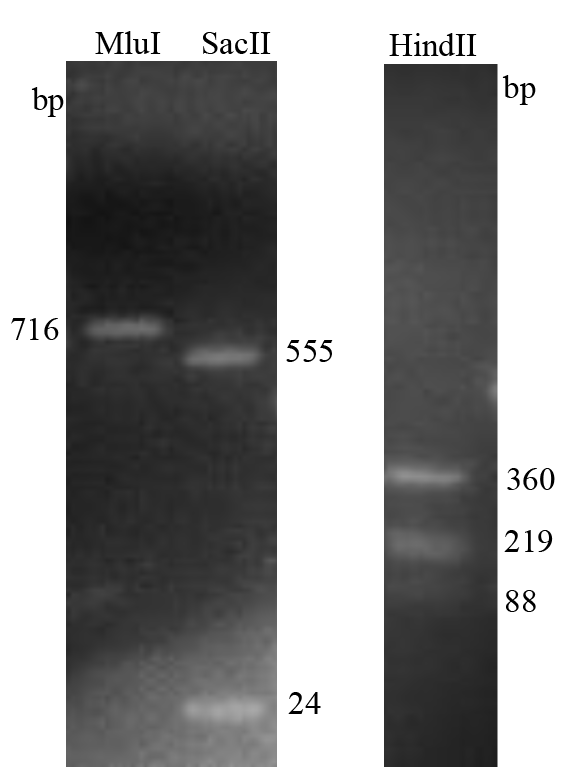

Supplement: Figure S1 [file peerj-07-7859-s001.png]
